# Novel Multi-Strain E3 Probiotic Formulation Improved Mental Health Symptoms and Sleep Quality in Hong Kong Chinese

**DOI:** 10.3390/nu15245037

**Published:** 2023-12-08

**Authors:** Helen Hoi Yin Chan, Pui Ling Kella Siu, Chi Tung Choy, Un Kei Chan, Junwei Zhou, Chi Ho Wong, Yuk Wai Lee, Ho Wang Chan, Joseph Chi Ching Tsui, Steven King Fan Loo, Stephen Kwok Wing Tsui

**Affiliations:** 1 Microbiome Research Centre, BioMed Laboratory Company Limited, Hong Kong, Chinakellasiu@biomed.com.hk (P.L.K.S.); yukichan@biomed.com.hk (U.K.C.); waynezhou@biomed.com.hk (J.Z.); howong@biomed.com.hk (C.H.W.); winnielee@biomed.com.hk (Y.W.L.); ivanchan@biomed.com.hk (H.W.C.); josephtsui@biomed.com.hk (J.C.C.T.); 2 Hong Kong Institute of Integrative Medicine, Faculty of Medicine, The Chinese University of Hong Kong, Hong Kong, China; 3 Dermatology Centre, CUHK Medical Centre, The Chinese University of Hong Kong, Hong Kong, China; 4 School of Biomedical Sciences, Faculty of Medicine, The Chinese University of Hong Kong, Hong Kong, China; 5 Centre for Microbial Genomics and Proteomics, The Chinese University of Hong Kong, Hong Kong, China; 6 Hong Kong Bioinformatics Centre, The Chinese University of Hong Kong, Hong Kong, China

**Keywords:** mental health, sleep quality, prebiotics, probiotics, postbiotics, gut microbiome, psychobiotics, metagenomics, *Bifidobacterium*, *Lactobacillus*

## Abstract

Mental health issues have emerged as a significant concern in public health, given their association with physical and psychological comorbidities and the resultant socioeconomic burdens. Recent studies have highlighted the interplay between gut microbes and brain functions through the gut–brain axis. To investigate this further, we conducted a targeted 16S rRNA sequencing and comprehensive bioinformatic analysis among Southern Chinese individuals to explore the role of the gut microbiome in depression, anxiety, and sleep disturbance. We analyzed the differences in the gut microbiome profile of 68 participants with sleep disturbance and mood symptoms before and after an 8-week course of a novel oral E3 multi-strain probiotics formula. The results revealed a significant improvement in subjective sleep quality (PSQI: mean 8.79 at baseline vs. 7.10 at week 8, *p* < 0.001), depressive symptoms (PHQ9: mean 6.17 at baseline vs. 4.76 at week 8, *p* < 0.001), and anxious symptoms (GAD7: mean 4.90 at baseline vs. 3.76 at week 8, *p* < 0.001). Additionally, there were notable differences in beta diversity (weighted UniFrac; *p* = 0.045) and increased Firmicutes/Bacteroidetes (F/B) ratio (*p* = 4 × 10^−4^) were observed in the gut microbiome analysis. Furthermore, the relative abundance of *Bifidobacterium bifidum* (*p* < 0.001), *Lactobacillus acidophilus* (*p* < 0.001), *Lactobacillus helveticus* (*p* < 0.001) and *Lactobacillus plantarum* (*p* < 0.001) were significantly increased after the 8-week probiotic supplementation. Our study suggests that the gut microbial landscape varies between responders and non-responders at multiple levels, including genera, species, functional, and network interaction. Notably, the use of probiotics in populations with depressive or anxious symptoms and poor sleeping quality remodeled the gut microbiome and demonstrated improved mood and sleep quality.

## 1. Introduction

Mental health disorders represent a significant public health issue, impacting individuals of all ages and leading to impaired daily functioning, diminished quality of life, and an increased likelihood of behavioral problems. A recent cohort study conducted amidst the COVID-19 pandemic highlighted the prevalence of depressive symptoms (PHQ-9 score ≥ 10) in 19% of the Hong Kong population, with 14% exhibiting anxiety (GAD score ≥ 10) [1]. Moreover, Hong Kong is recognized as one of the most sleep-deprived regions worldwide, with up to 48% of its population reporting sleep-related issues according to the Population Health Survey conducted by the Department of Health. These statistics are alarming, especially considering that 74% of individuals with common mental disorders do not seek professional help [2]. Among various mental disorders, it is worth noting that young people aged 15–24 years experiencing major depression episodes have the lowest service utilization rate at 16.7%, which can be attributed to the high cost of psychiatric service [3], indicating a significant gap in access to appropriate care and support for this vulnerable population.

Despite the widespread impact of mental health issues, they are often misunderstood and receive less immediate attention than physical illnesses. The intricate underlying pathophysiology and neurobiological mechanisms of mental health disorders present a significant challenge in managing depressive and anxiety symptoms and insomnia. The role of the microbiome in mental health through microbiota–gut–brain axis has become an emerging area of study. The gut and brain communicate through various pathways, including vagus nerve (VN), the autonomic nervous system (ANS) in the spinal cord, and bidirectional communication within the enteric nervous system [4,5]. The vagus nerve, comprising both afferent and efferent branches, serves as a vital connection between the brain and gut. The presence of gastrointestinal vagal afferents and the expression of mechanoreceptors and chemoreceptors on these afferents enable the detection and response to signals from the gut microbiota, influencing stress reactivity and depression- or anxiety-like behaviors [6,7,8]. Interestingly, gut the microbiota also has the capability to synthesize neurotransmitters such as GABA, serotonin (5-HT), glutamate, and dopamine, in which their dysregulation is associated with anxiety and depression and affects sleep quality [9,10,11,12,13]. Notably, over 90% of the body’s 5-HT is synthesized in the gut [14,15]. For instance, *Lactobacillus plantarum*, *Lactococcus lactis* subsp. *cremoris*, and *Streptococcus thermophilus* are known to produce 5-HT, while *Bifidobacterium infantis* increases tryptophan, a precursor of 5-HT [16]. In addition, *Lactobacillus rhamnosus* can also directly affect GABA receptor expression in CNS, affecting anxiety and depressive behaviors [17]. Consequently, microbiota likely influence brain function and behavior significantly by altering the concentrations of neurotransmitters in the brain, playing a critical role in regulating depression, anxiety-like behaviors, and sleep–wake patterns [18,19,20].

Psychobiotics are psychotropic live organisms that interact with commensal gut bacteria with the ability to release neuroactive substances and act on the brain–gut axis [21], conferring mental health benefits such as anxiolytic and antidepressant effects. The *Bifidobacterium* and *Lactobacillus* families mentioned above are examples of psychobiotics that are associated with GABA, serotonin, and acetylcholine expression. A recent meta-analysis of double-blind, randomized, placebo-controlled trials involving a total of 1901 participants with depressive symptoms suggested that probiotics could be a promising strategy for improving the severity of depression and anxiety [22,23]. In addition, prebiotic, such as fructooligosaccharide (FOS) and galatooligosaccharide (GOS), administration also demonstrated anxiolytic and antidepressant effects with reduced stress-induced corticosterone in mice [24]. Considering challenges in mental health disorders, it is not uncommon for patients to seek alternative methods of support, such as over-the-counter supplementation. Due to their availability as over-the-counter supplements, probiotics offer individuals a convenient and accessible means of potentially improving their mental health.

In this study, our primary objective was to evaluate the efficacy of an 8-week intervention using a novel multi-strain probiotic mixture intervention in participants with depressive and anxious symptoms and sleep disturbance. We also investigated the gut microbiome evolution through 16S rRNA sequencing before and after the intervention. Our ultimate goal was to assess and enhance the clinical effectiveness of probiotics as an intervention for individuals with mild-to-moderate anxiety and depressive symptoms or sleep disturbance. By optimizing the probiotic formulation, we aimed to provide individuals with a self-care option that can potentially contribute to their mental well-being.

## 2. Materials and Methods

### 2.1. Subject Recruitment and Study Design

Subjects with sleep disturbance and mood symptoms were recruited through the collaboration between Hong Kong Society of Gut Microbiome (HKSGM) and local patient groups from March 2021 to February 2022. To be included in this study, all participants had to be (1) above 18 years of age, and (2) provide signed informed consent. Subjects with specific conditions including (1) adverse reactions to probiotics, (2) recent bacterial infections, (3) pregnancy, (4) pre-existing medical conditions, such as cardiovascular, liver, or renal diseases, or diabetes, (5) recent use of certain medications, including oral corticosteroids/antibiotics, immunosuppressive or any oral herbal medicines, and (6) use of anti-coagulant/anti-platelet drugs in the past month were not recruited or were excluded. The Pittsburgh Sleep Quality Index (PSQI), General Anxiety Disorder-7 (GAD-7), and Patient Health Questionnaire-9 (PHQ-9) were assessed with fecal samples collected at baseline and week 8 after receiving oral multi-strain probiotics. A total of 7 components were addressed in PSQI, including sleep duration, sleep disturbance, sleep latency, daytime dysfunction due to sleepiness, sleep efficiency, overall sleep quality, and sleep medication use. PHQ-9 is a useful tool for depression screening and has been validated in Chinese population [25]. A participant was identified as a “responder” if there was a reduction of at least 3 points in the PSQI score, a 20% reduction in the PHQ-9 score, or a reduction in the GAD-7 score.

This research study was conducted in compliance with the Declaration of Helsinki guidelines and was approved by the Research Ethics Committee of the Hong Kong Doctors Union (protocol number: HKSGM-2020AD-Study-protocol-v1-20220211).

### 2.2. Library Preparation and 16S rRNA Sequencing

Stool samples were collected before and after 8 weeks of probiotic supplementation, which were then homogenized in the DNA Hypoosmotic stabilizer (BioMed Technology Holdings Limited, Hong Kong, China). The samples were then beaten with glass beads (425–600 µm) (Sigma-Aldrich, Saint Louis, MO, USA) for an hour, following the manufacturer’s instructions. Microbial DNA was extracted from the stool samples using the DNeasy Blood & Tissue Kit (Qiagen, Hilden, Germany). The extracted DNA concentration of each sample was measured using a Qubit™ dsDNA HS Assay Kit (Life Technologies, Carlsbad, CA, USA) with Qubit 3 Fluorometer (Thermo Fisher Scientific, Waltham, MA, USA).

An amplicon library was constructed using the primer pair 515F (5′-GTGCCAGCMGCCGCGG-3′)/907R (5′-CCGTC-AATTTCMTTTRAGTTT-3′), targeting the V4-V5 hypervariable of 16S rRNA genes. This library also included the adapter sequences, multiplex identifier tags, and library keys. The sequencing of the 16S rRNA gene was performed using the Illumina MiSeq platform (Illumina, Inc., San Diego, CA, USA) following the protocols of original Earth Microbiome Project [26]. After demultiplexing, the index barcodes and adaptor sequences were removed from the paired-end reads for subsequent analysis.

### 2.3. Probiotic Mixture

A daily capsule of a novel E3 probiotic formula, developed by BioMed Microbiome Research Centre (BioMed Laboratory Company Limited, Hong Kong, China), was given to all participants. The formula consisted of a blend of 8 highly effective gastro-resistant probiotic strains (with a minimum of 2 × 10^11^ CFU/capsule at the time of production), prebiotics including inulin and oligosaccharides powder, and postbiotic extract from *Lactobacillus plantarum*. The probiotic mixture was made up of *Lactobacillus acidophilus* GKA7, *Lactobacillus casei* GKC1, *Lactobacillus helveticus* GKS3, *Lactobacillus plantarum* GKM3, *Bifidobacterium bifidum* GKB2, and *Bifidobacterium longum* GKL7.

### 2.4. Bioinformatics Analysis

Only subjects with (1) gut microbiome data available before and after taking oral multi-strain probiotic supplement for 8 weeks and (2) Pittsburgh Sleep Quality Index (PSQI), General Anxiety Disorder-7 (GAD-7), and Patient Health Questionnaire-9 (PHQ-9) scores available before and after taking oral multi-strain probiotic supplement were included in this analysis.

QIIME2-2023.5, a plugin-based platform that integrates various microbiome analysis algorithms and tools [27], was used to analyze the microbiome bioinformatics data for this study. Analysis of Compositions of Microbiomes with Bias Correction (ANCOM-BC) analysis was performed to investigate differential abundance in gut microbiome [28].

### 2.5. Statistical Analysis

R (v4.3.1) packages used in this study include: qiime2R (v0.99.6), ggplot2 (v3.3.5), ggpubr (v0.6.0), and R (v4.3.1).

## 3. Results

### 3.1. Study Cohort

Seventy-six participants with sleep disturbance and mood symptoms were recruited initially in this prospective cohort study. However, eight participants were excluded from the analysis due to incomplete fecal sample collection or loss to follow up. Before and after the oral administration of the probiotic mixture, their sleep quality was evaluated by the Pittsburgh Sleep Quality Index (PSQI), while their anxiety and depression statuses were evaluated using the General Anxiety Disorder-7 (GAD-7) and Patient Health Questionnaire-9 (PHQ-9), respectively. The questionnaire also encompassed various factors that might influence the gut microbiome, such as age, sex, body mass index (BMI), presence of allergies, and history of probiotic and antibiotic use. Among the 68 participants included in the analysis, 9 of them exhibited moderate-to-severe anxiety symptoms, with a GAD-7 score higher than 10. Sixteen participants demonstrated moderate to severe depressive symptoms, with a PHQ-9 score higher than 10. Furthermore, 55 of them experienced poor sleep quality, with a PSQI score higher than 5 at baseline. These findings indicate a high prevalence of anxiety (13.24%), depression (23.53%), and sleep disturbance (80.89%) within the study population.

### 3.2. Symptomatic Improvements in Sleep Quality and Depressive and Anxious Symptoms with 8-Week Probiotic Intervention

An 8-week course of a probiotic mixture, incorporating multiple strains of *Bifidobacterium* and *Lactobacillus*, along with prebiotic inulin and oligosaccharides, as well as postbiotics extract from *Lactobacillus plantarum*, was orally administrated by the participants. Out of the total 68 participants, a total of 46 participants demonstrated a significant response after 8 weeks of probiotic treatment compared to baseline. The intervention resulted in significant improvements in sleep quality, depressive symptoms, and anxious symptoms. Sleep quality and depressive and anxious symptoms were assessed via self-report questionnaire by participants.

The PSQI score, used to evaluate the sleep quality of participants decreased from a mean score of 8.79 at baseline to 7.10 at week 8 (*p* < 0.001, Wilcoxon), indicating a significant enhancement in sleep quality in response to the probiotic intervention. Notably, a higher percentage of participants with poor sleep quality at baseline (70.9%; *p* < 0.01) demonstrated significant improvement compared to participants with good sleep quality at baseline (23.1%; *p* = 0.7103).

**Figure 1:**
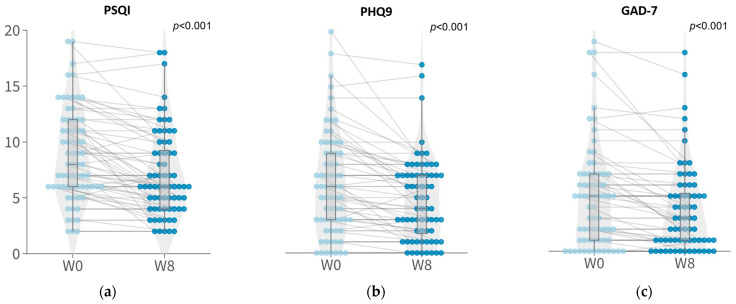
Boxplots of sleep quality, depressive symptom, and anxious symptom scores of participants at week 0 and week 8. (**a**) Pittsburgh Sleep Quality (PSQI); (**b**) Patient Health Questionnaire-9 (PHQ9); (**c**) General Anxiety Disorder (GAD7).

The severity of depressive symptoms, assessed using the PHQ-9, also showed a significant reduction after the 8-week probiotic intervention from a mean score of 6.17 at baseline to 4.76 at week 8 (*p* < 0.001, Wilcoxon). Among the 16 participants with depressive symptoms at baseline, 75% of them showed improvement in depressive symptoms with at least a 20% reduction in their PHQ-9 scores. A subset of participants (30.77%) with minimal to mild depressive symptoms at baseline also showed a response. Additionally, anxiety symptoms, measured using GAD-7, significantly decreased after the 8-week probiotics treatment from a mean score of 4.90 at baseline to 3.76 at week 8 (*p* < 0.001, Wilcoxon), and significantly improved after the 8-week probiotic treatment (Figure 1). Among the nine participants with anxiety symptoms at baseline, 55.56% showed symptomatic improvement, with a reduction in their GAD-7 scores. Similarly, 35.59% of participants with minimal to mild anxiety symptoms showed a reduction in their GAD-7 scores.

The demographic and disease characteristics shown in Table 1 did not demonstrate statistically significant differences between responders and non-responders in terms of age (*p* = 0.5027), sex (*p* = 0.7940), and BMI (*p* = 0.7188). Therefore, no adjustment was performed for these characteristics during the subsequent analysis. Significant differences in ΔGAD7, ΔPHQ9, and ΔPSQI were observed between the responder and non-responder group (ΔGAD7: *p* < 0.001; ΔPHQ9: *p* < 0.001; ΔPSQI: *p* < 0.001, Wilcoxon).

### 3.3. Improvement in the Relative Abundance of Probiotic Species after 8-Week Probiotic Intake

**Figure 2:**
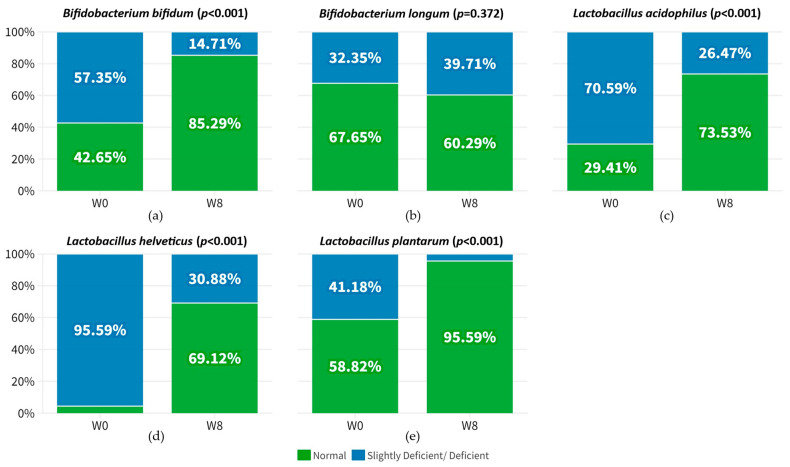
Proportion of participants who achieved normal relative abundance (green) of respective bacterial targets at week 0 and at week 8. (**a**) *Bifidobacterium bifidum*; (**b**) *Bifidobacterium longum*; (**c**) *Lactobacillus acidophilus*; (**d**) *Lactobacillus helveticus*; (**e**) *Lactobacillus plantarum*.

Improved gut composition was observed in participants after an 8-week course of multi-strain probiotics. We examined the changes in the participants’ gut microbiome profile after an 8-week intake of probiotics, comparing it to their baseline profile. This analysis aimed to assess the extent of alteration in the gut microbiome, as it may serve as an indicator of the individuals’ responsiveness to probiotics. Notably, the relative abundance of probiotic species *Bifidobacterium bifidum* (ΔCt −19.48%; *p* < 0.001), *Lactobacillus acidophilus* (ΔCt −13.06%; *p* < 0.001), *Lactobacillus helveticus* (ΔCt −21.39%; *p* < 0.001), and *Lactobacillus plantarum* (ΔCt −42.49%; *p* < 0.001) were significantly upregulated after 8 weeks of probiotics in participants (Figure 2 and Figure S1). These findings suggest that the consumption of probiotic mixtures rich in *Lactobacillus* and *Bifidobacterium* contributed to the heightened abundance of these species. In particular, the relative abundance of *Bifidobacterium bifidum*, *Lactobacillus acidophilus*, *Lactobacillus helveticus*, and *Lactobacillus plantarum* significantly increased within the poor sleeper and depression groups following the probiotic treatment, which may have contributed to the notable improvement in sleep quality and depressive symptoms (Figures S2 and S4). Similarly, the relative abundance of *Lactobacillus acidophilus*, *Lactobacillus helveticus*, and *Lactobacillus plantarum* significantly increased in anxiety group (Figure S3), potentially contributing to the improvement of anxiety symptoms. Consequently, these results indicate favorable changes in the gut microbiome composition resulting from the probiotic intervention, and highlight the importance of specific probiotic strains.

**Figure 3:**
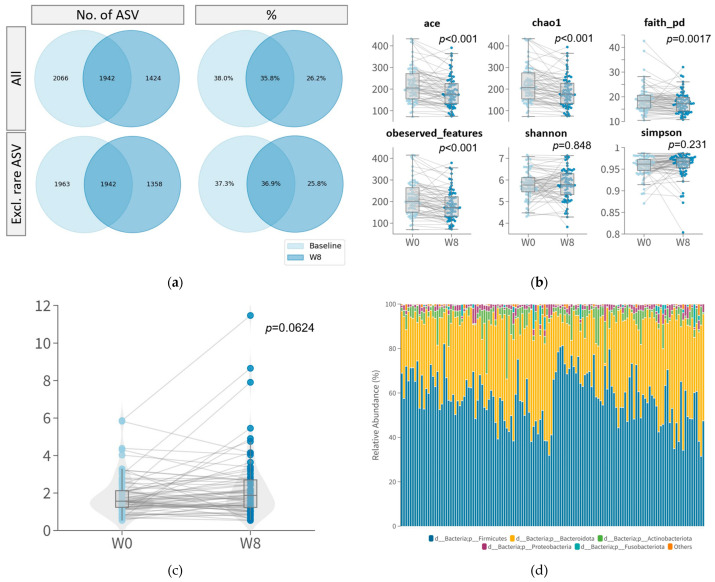
Gut microbiome composition of participants at week 0 and at week 8. (**a**) Venn diagram of number (**left**) and percentage (**right**) of all ASVs (**top**) and with rare ASVs excluded (**bottom**) at week 0 and at week 8. ASVs with only one count in samples were defined as rare ASVs; (**b**) alpha diversity; (**c**) boxplot of Firmicutes/Bacteroides (F/B) ratio; (**d**) relative abundance of phyla.

The richness and diversity of the gut microbiome community improved after 8 weeks of probiotic intervention. A total of 5432 unique amplicon sequence variants (ASVs) were identified. Among these, 169 ASVs were classified as rare, with only one count in the samples. After alignment, these ASVs were further assigned to 14 phyla, 22 classes, 54 orders, 106 families, 289 genera, and 703 species. At baseline, 3618 ASVs were identified, while at week 8, 3366 ASVs were identified, with only 1942 ASVs shared before and after the 8-week course of probiotics (Figure 3a). Among these identified ASVs, there was no significant difference in the abundance between responders and non-responders with respect to their PSQI, GAD-7, or PHQ-9 scores.

After 8 weeks of probiotic supplementation, there was a weak decrease in alpha-diversity measures but an increased Firmicutes/Bacteroides (F/B) ratio. Four out of six measures of alpha-diversity, including ACE (*p* < 0.001), Chao1 (*p* < 0.001), Faith’s-PD (*p* = 0.001), and observed features (*p* < 0.001), showed a significant reduction after 8-week probiotic supplementation (Figure 3b). This indicates a less diverse microbial community or a shift in the dominant microbial species within the community in response to the probiotics. However, no significant difference in alpha diversity between responders and non-responders at week 0 or week 8 was observed.

Regarding taxonomy, the Firmicutes/Bacteroides (F/B) ratio marginally increased (*p* = 0.0624, Wilcoxon Signed Rank) after probiotic supplementation. At the phylum level, *Firmicutes*, *Bacteroidota*, *Actinobacteriota*, *Proteobacteria*, and *Fusobacteriota* dominated the intestinal microbiota in our participants. No significant changes were observed after 8-week probiotic treatment and between responders and non-responders.

**Figure 4:**
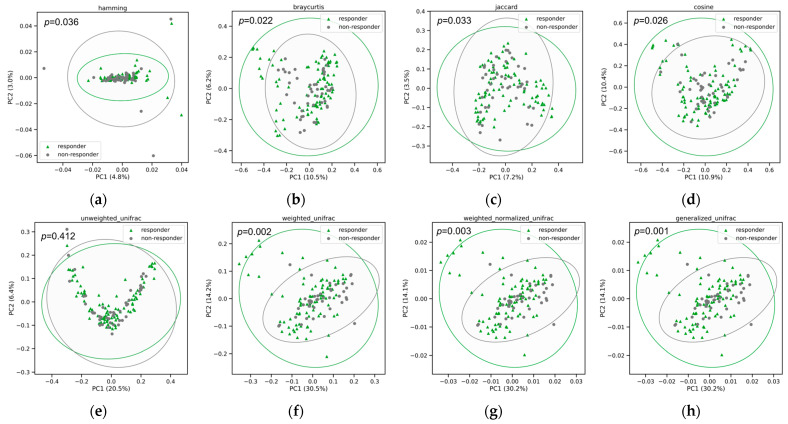
Principal coordinate analysis with 99% confidence ellipse of responder and non-responder at baseline based on (**a**) Hamming; (**b**) Bray–Curtis; (**c**) Jaccard; (**d**) Cosine; (**e**) unweighted UniFrac; (**f**) weighted UniFrac; (**g**) weighted normalized UniFrac; and (**h**) generalized UniFrac distance.

To investigate variations in the composition and organismal structure of the intestinal microbiota, we conducted a principal coordinates analysis (PCoA) using weighted UniFrac distances derived from 16S rRNA sequence profiles at the ASVs level. Significant differences were observed in seven out of eight beta diversities between responders and non-responders, indicating composition differences between the groups. However, there was no significant interaction between the timepoint and response. In addition to individual heterogeneity, antibiotics and response to probiotics explained approximately 3–5% of the variation in the gut microbiome in general. The results of the PCoA analysis indicated a clustering pattern for intestinal samples from responders and non-responders, suggesting that the gut microbiome composition of responders was significantly different from non-responders at baseline based on Hamming (*p* = 0.036), Bray–Curtis (*p* = 0.022), Jaccard (*p* = 0.033), Cosine, (*p* = 0.026), weighted UniFrac (*p* = 0.002), normalized UniFrac (*p* = 0.003), and generalized UniFrac distance (*p* = 0.001) (Figure 4). These results were further supported by the weighted Unifrac distances between responders and non-responders according to its kernel density distribution profile (Figure 4f).

When stratifying by symptoms (Table S2), significant differences in beta diversity were observed between individuals with good and poor sleep quality (Bray–Curtis: *p* = 0.028; Hamming: *p* = 0.009; Jaccard: *p* = 0.012). Significant beta diversity difference was also observed between individuals in the moderate anxiety and no-to-minimal anxiety group (Jaccard: *p* = 0.010). However, no significant difference was observed between depressive and no-to-minimal depressive individuals (Figures S5–S8).

### 3.4. Functional Abundance and Pathways

To explore the functional differences in intestinal microbiota between responders and non-responders, both before and after probiotic intervention, we utilized PICRUSt2 for the silico inference of functional abundance of MetaCyc pathways, guided by the LefSe algorithm.

Our analysis identified 22 discriminative features with an absolute LDA larger than two. Among these features, 14 MetaCyc pathways exhibited differential abundance in the responder group, while 8 MetaCyc pathways exhibited differential abundance in the non-responder group at week 8 (Table S3). The majority of features overrepresented in the responder group were associated with the biosynthesis pathways, followed by the degradation or metabolism of vitamins, such as thiamine and queuosine, sugar nucleotides, such as GDP-mannose and CMP-3-deoxy-D-*manno*-octulosonate, and the essential amino acid L-Tryptophan. Note that tryptophan is a precursor of serotonin, and lower thiamine levels have been correlated with anxiety and depressive symptoms [29,30]. The enriched biosynthesis pathway abundance for L-tryptophan and thiamine may potentially contribute to the observed symptomatic improvements after 8 weeks of probiotic intervention.

**Figure 5:**
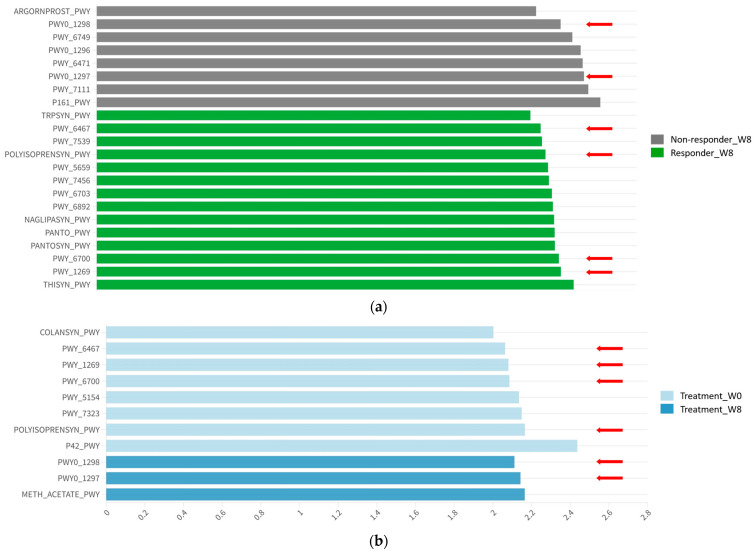
Predicted MetaCyc pathways’ abundance. Pathways reported in (**a**,**b**) are indicated by a red arrow. (**a**) Log LDA score of MetaCyc pathways with differential abundance between responder and non-responder group at week 8; (**b**) log LDA score of MetaCyc pathways with differential abundance between participants at week 0 and week 8.

**Figure 6:**
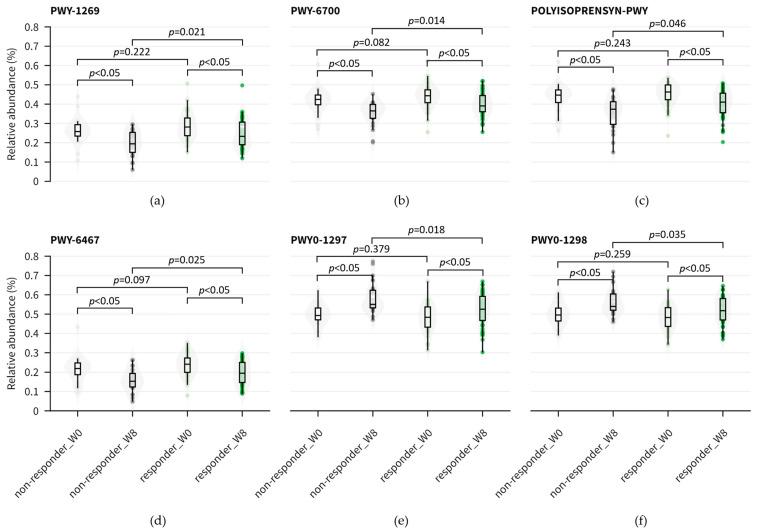
Boxplot of relative abundance of MetaCyc pathways indicated by red arrow in Figure 5. (**a**) CMP-3-deoxy-D-*manno*-octulosonate biosynthesis, PWY-1269. (**b**) Queuosine biosynthesis I (de novo), PWY-6700. (**c**) Polyisoprenoid biosynthesis (*E. coli*), POLYISOPRENSYN-PWY. (**d**) Kdo transfer to lipid IV_A_ (*Chlamydia*), PWY-6467. (**e**) Superpathway of purine deoxyribonucleoside degradation, PWY0-1297. (**f**) Superpathway of pyrimidine deoxyribonucleoside degradation, PWY0-1298.

Additionally, we observed that several pathways, including CMP-3-deoxy-D-*manno*-octulosonate biosynthesis (PWY-1269), de novo Queuosine biosynthesis I (PWY-6700), Polyisoprenoid biosynthesis (*E. coli*) (POLYISOPRENSYN-PWY), and Kdo transfer to lipid IV_A_ (*Chlamydia*) (PWY-6467) exhibited similar levels of abundance at baseline between responders and non-responders (Figure 5a). However, following probiotic intervention, these pathways showed a significant reduction in abundance in both groups (*p* < 0.05) (Figure 6a–d). On the other hand, the non-responder group exhibited an abundance of pathways primarily involved in degradation processes, including arginine, pyruvate, acetylene, purine deoxyribonucleosides, and pyrimidine.

When comparing the effects of probiotic intervention, eight pathways were shown to be differentially abundant prior to the intervention, while only three pathways showed differential abundance post intervention (Table S4, Figure 5b). The relative abundance of superpathways of purine deoxyribonucleoside degradation (PWY0-1297) and pyrimidine deoxyribonucleoside degradation (PWY0-1298) were significantly upregulated after 8-week probiotic intervention in both responders and non-responders (*p* < 0.05) (Figure 6e,f). The downregulation of purine or pyrimidine metabolism was associated with depression [31,32] and sleep disorders [33]. Therefore, the upregulated metabolic pathways mediated by probiotics could potentially contribute to the reduction in anxiety and depressive symptoms and improvement in observed sleep quality.

## 4. Discussion

Our prospective cohort study provide evidence of the beneficial effects of an 8-week intervention with a novel multi-strain E3 probiotic containing prebiotic, probiotics, and postbiotics on symptoms of anxiety, depression, and poor sleep quality, according to the subjective measurements of GAD7, PHQ9, and PSQI, respectively. Significant improvements were observed in these outcome measurements, especially in participants with depressive symptoms and poor sleep quality at baseline, suggesting that probiotics may positively impact mental health and sleep quality.

Furthermore, the study revealed the effects of probiotic intervention in gut microbiome composition. The observed upregulation of specific probiotic species, including *Bifidobacterium bifidum*, *Lactobacillus acidophilus*, *Lactobacillus helveticus*, and *Lactobacillus plantarum*, suggests their potential role in the observed symptomatic improvements. These findings align with previous studies demonstrating the beneficial effects of probiotic combinations containing *Lactobacillus* and *Bifidobacterium* on anxiety- and depression-related behaviors [34,35,36,37]. Evidence suggests that *Lactobacillus* species, such as *L. rhamnosus*, *L. acidophilus*, and *L. plantarum* can influence the production and metabolism of serotonin (5-HT) and maintain its levels in the gastrointestinal tract [16,18,19]. Moreover, the administration of *L. rhamnosus* in mice has been shown to increase brain glutamate and GABA concentrations, leading to reductions in anxiety- and depression-related behaviors [17,38]. In this study, the current probiotic formulation, which included these strains, led to an increase in the psychobiotic species richness and beta diversity increased, alongside with improvements in symptoms. Particularly, participants with baseline anxiety, depressive-like symptoms, and poor sleep quality experienced notable benefits. These findings highlight the potential of the probiotic intervention to positively influence the gut microbiome, with a favorable shift in the gut microbiota composition and a beneficial role in promoting mental well-being.

Previous analyses have explored the complex interplay between probiotics, the gut microbiota, and mental health. At the functional pathway and network level, enriched pathways related to the biosynthesis of thiamine and 5-HT precursor L-tryptophan were observed in the responder group, as well as an enriched metabolism of purine and pyrimidine after 8-week probiotic treatment. Altered levels of thiamine [29,30,39], serotonin [9,15], and metabolites of purine and pyrimidine [32] have been associated with the emergence of anxiety, depression, and sleep disorders. The ingestion of a mixture of *Lactobacillus* and *Bifidobacterium* might promote thiamine and 5-HT synthesis in the gut, leading to enhanced neurotransmission, thereby improving symptoms. These effects could potentially be attributed to the influence on serotonergic or GABAergic neurotransmission and gut–brain vagus nerve activity. Further clinical investigations are warranted to validate the impact of the current probiotic formulation on the levels of neurotransmitters such as GABA, dopamine, and serotonin, as well as to compare the effects of this probiotic formulation with a placebo.

It is important to note that the inclusion of prebiotics in the formulation, including galacto-oligosaccharides (GOS) and fructo-oligosaccharides (FOS) also contributed to the psychobiotic properties. Prebiotic consumption not only serves as a nourishing source for *Bifidobacterium* and *Lactobacilli*, but also leads to an elevation in BDNF level and NR1 mRNA expression in the hippocampus in animal studies [40] and reduced waking cortisol response [41] with anxiolytic and antidepressant effects in human studies [42]. These findings contribute to our understanding of the intricate interactions between the gut microbiome, probiotics, and host well-being, and warrant further research, especially randomized placebo-controlled clinical study, to validate and explore these functional and metabolomics implications of probiotics therapy in greater detail.

## 5. Conclusions

In summary, this novel E3 formulation incorporating prebiotics, probiotics, and postbiotics was noted to exert a favorable impact on mood symptoms and sleep quality. The observed changes in the gut microbiome composition between responders and non-responders, before and after 8-week supplementation, shed light on the microbial dynamics and potential implications for mental health in the context of probiotic interventions. We have also proposed potential mechanisms that could explain the observed improvements in mental health and sleep quality. These findings offer valuable insights into the potential psychological benefits of probiotics, expanding our understanding of their therapeutic potential.

## Figures and Tables

**Table 1:** Baseline characteristics of participants.

	Patients (No.)		
Variable	Responder ^5^ (*n* = 46)	Non-Responder (*n* = 22)	*p* Value
**Characteristics**			
Sex, No. (%)			
Male	17 (37.0)	9 (40.9)	0.7940
Female	29 (63.0)	13 (59.1)	
Age, median [range], y	52.0 [21–79]	52.5 [22–67]	0.5027
BMI ^1^, mean (SD)	22.6 (4.1)	22.8 (3.5)	0.7188
Allergy ever, No. (%)	18 (39.1)	5 (22.7)	0.2735
ΔGAD7, mean (SD) ^2^	−1.7 (2.4)	0.1 (0.3)	<0.001
Minimal or No anxiety, No. (%)	40 (87.0)	19 (86.4)	
Anxiety Symptoms, No. (%)	6 (13.0)	3 (13.6)	
ΔPHQ9, mean (SD) ^3^	−2.1 (2.6)	0.05 (0.4)	<0.001
Minimal or No Depressive, No. (%)	33 (71.7)	19 (86.4)	
Depressive Symptoms, No. (%)	13 (28.3)	3 (13.6)	
ΔPSQI, mean (SD) ^4^	−2.4 (2.3)	−0.1 (1.2)	<0.001
Normal Sleeper, No. (%)	10 (21.7)	3 (13.6)	
Poor Sleeper, No. (%)	36 (78.3)	19 (86.4)	

^1^ Body Mass Index. ^2^ General Anxiety Disorder-7. ^3^ Patient Health Questionnaire-9. ^4^ Pittsburgh Sleep Quality Index. ^5^ Either ΔPSQI smaller than −3, ΔPHQ9 smaller than −20% or ΔGAD7 smaller than −20% was regarded as responders.

## Data Availability

The raw sequence data are available in NCBI (PRJNA1037285). Due to the restriction of consent and sensitivity, the metadata and qPCR data are available upon reasonable request made to the corresponding authors.

## Supplementary Materials

The following supporting information can be downloaded at: https://www.mdpi.com/article/10.3390/nu15245037/s1.
