# Supplementary material for: Novel Multi-Strain E3 Probiotic Formulation Improved Mental Health Symptoms and Sleep Quality in Hong Kong Chinese
Source: Nutrients. 2023 Dec 8;15(24):5037. doi: 10.3390/nu15245037 (PMC10745623; doi:10.3390/nu15245037)
Supplement: Supplementary file 1 [file nutrients-15-05037-s001.zip › Figures and Tables caption_20231205.pdf]

Supplementary Figure S1. Gut microbiome profiles of participants at week 0 and at week 8. (a) ΔCt of *Bifidobacterium bifidum*, (b) ΔCt of *Bifidobacterium longum*, (c) ΔCt of *Lactobacillus acidophilus*, (d) ΔCt of *Lactobacillus helveticus*, and (e) ΔCt of *Lactobacillus plantarum*.

Supplementary Figure S2. Gut microbiome profiles of participants with poor sleep quality at week 0 and at week 8. (a) ΔCt of *Bifidobacterium bifidum*, (b) ΔCt of *Bifidobacterium longum*, (c) ΔCt of *Lactobacillus acidophilus*, (d) ΔCt of *Lactobacillus helveticus*, and (e) ΔCt of *Lactobacillus plantarum*.

Supplementary Figure S3. Gut microbiome profiles of participants in anxiety group at week 0 and at week 8. (a) ΔCt of *Bifidobacterium bifidum*, (b) ΔCt of *Bifidobacterium longum*, (c) ΔCt of *Lactobacillus acidophilus*, (d) ΔCt of *Lactobacillus helveticus*, and (e) ΔCt of *Lactobacillus plantarum*.

Supplementary Figure S3. Gut microbiome profiles of participants in depressive group at week 0 and at week 8. (a) ΔCt of *Bifidobacterium bifidum*, (b) ΔCt of *Bifidobacterium longum*, (c) ΔCt of *Lactobacillus acidophilus*, (d) ΔCt of *Lactobacillus helveticus*, and (e) ΔCt of *Lactobacillus plantarum*.

Supplementary Figure S5. Boxplot of alpha diversity of responder and non-responder at week 0 based on (a) ACE Index, (b) Chao1 Index, (c) Faith's Phylogenetic Diversity, (d) the Observed OTUs, (e) Shannon Diversity Index, and (f) Simpson Index.

Supplementary Figure S6. Boxplot of alpha diversity of responder and non-responder at week 8 based on (a) ACE Index, (b) Chao1 Index, (c) Faith's Phylogenetic Diversity, (d) the Observed OTUs, (e) Shannon Diversity Index, and (f) Simpson Index.

Supplementary Figure S7. Boxplot of alpha diversity of responder at week 0 and at week 8 based on (a) ACE Index, (b) Chao1 Index, (c) Faith's Phylogenetic Diversity, (d) the Observed OTUs, (e) Shannon Diversity Index, and (f) Simpson Index.

Supplementary Figure S8. Boxplot of alpha diversity of non-responder at week 0 and at week 8 based on (a) ACE Index, (b) Chao1 Index, (c) Faith's Phylogenetic Diversity, (d) the Observed OTUs, (e) Shannon Diversity Index, and (f) Simpson Index.

Supplementary Figure S9. Pathway diagram of pathways in Figure S6 from MetaCyc. (a) CMP-3-deoxy-D-*manno*-octulosonate biosynthesis, PWY-1269. (b) Queuosine biosynthesis I (*de novo*), PWY-6700. (c) Polyisoprenoid biosynthesis (*E. coli*), POLYISOPRENSYN-PWY. (d) Kdo transfer to lipid IVA (*Chlamydia*), PWY-6467. (e) Superpathway of purine deoxyribonucleosides degradation, PWY0-1297. (f) Superpathway of pyrimidine deoxyribonucleosides degradation, PWY0-1298.

Supplementary Table S1. Baseline demographic characteristics of participants.

Supplementary Table S2. ADNOIS beta diversity of participants based on Hamming, Bray-Curtis, Jaccard, Cosine, Unweighted UniFrac, Weighted UniFrac, Weighted normalized UniFrac, and Generalized UniFrac distances.

Supplementary Table S3. Differentially abundant MetaCyc pathways between responder and non-responder group at week 8 and its corresponding LDA score, and *p* value inferred by PICRUSt2 and LefSe.

Supplementary Table S4. Differentially abundant MetaCyc pathways of participants at week 0 and at week 8 and its corresponding LDA score, and *p* value inferred by PICRUSt2 and LefSe.
