# Supplementary material for: Novel Multi-Strain E3 Probiotic Formulation Improved Mental Health Symptoms and Sleep Quality in Hong Kong Chinese
Source: Nutrients. 2023 Dec 8;15(24):5037. doi: 10.3390/nu15245037 (PMC10745623; doi:10.3390/nu15245037)

Supplementary Figure S1

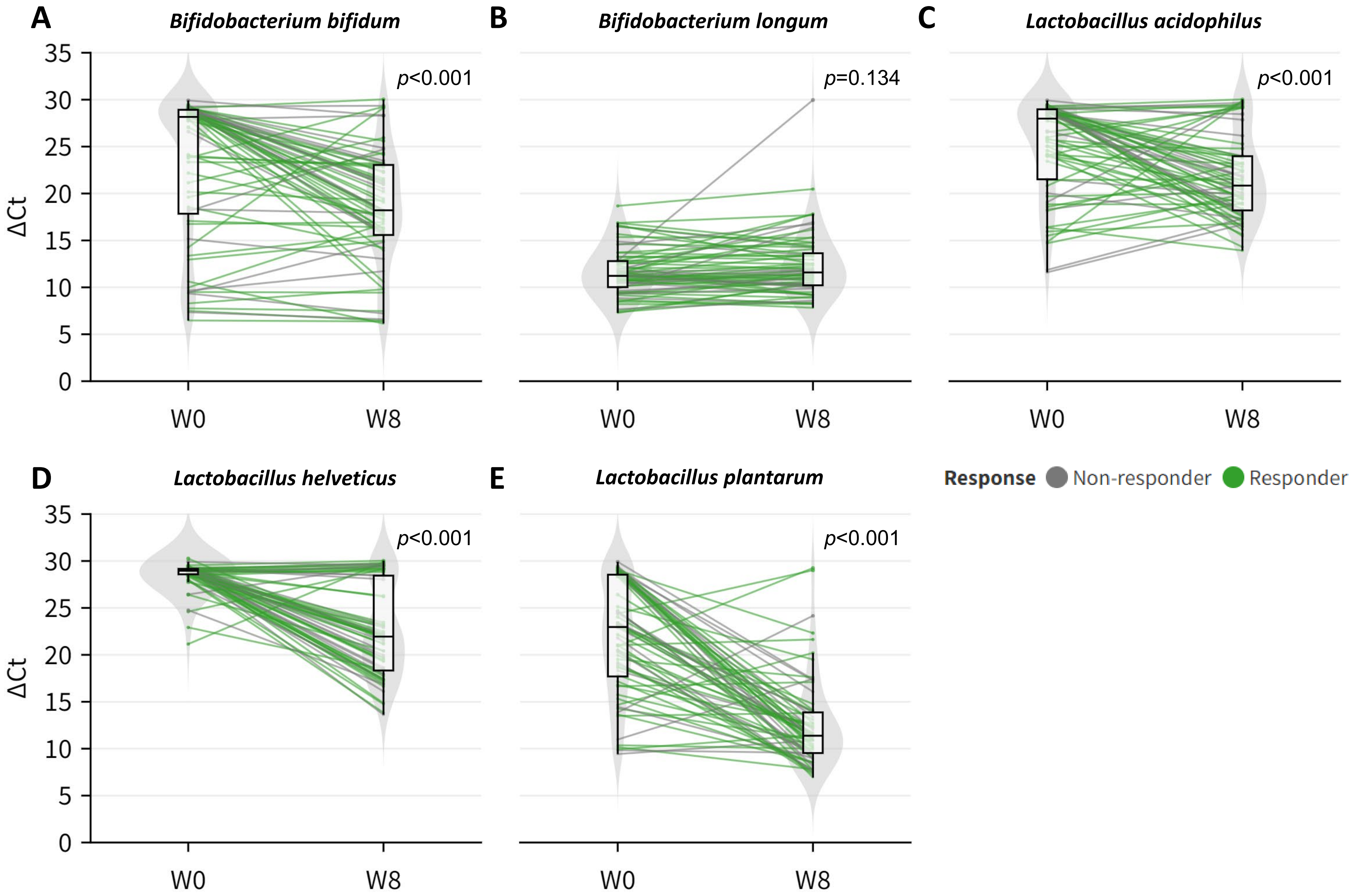

Supplementary Figure S2

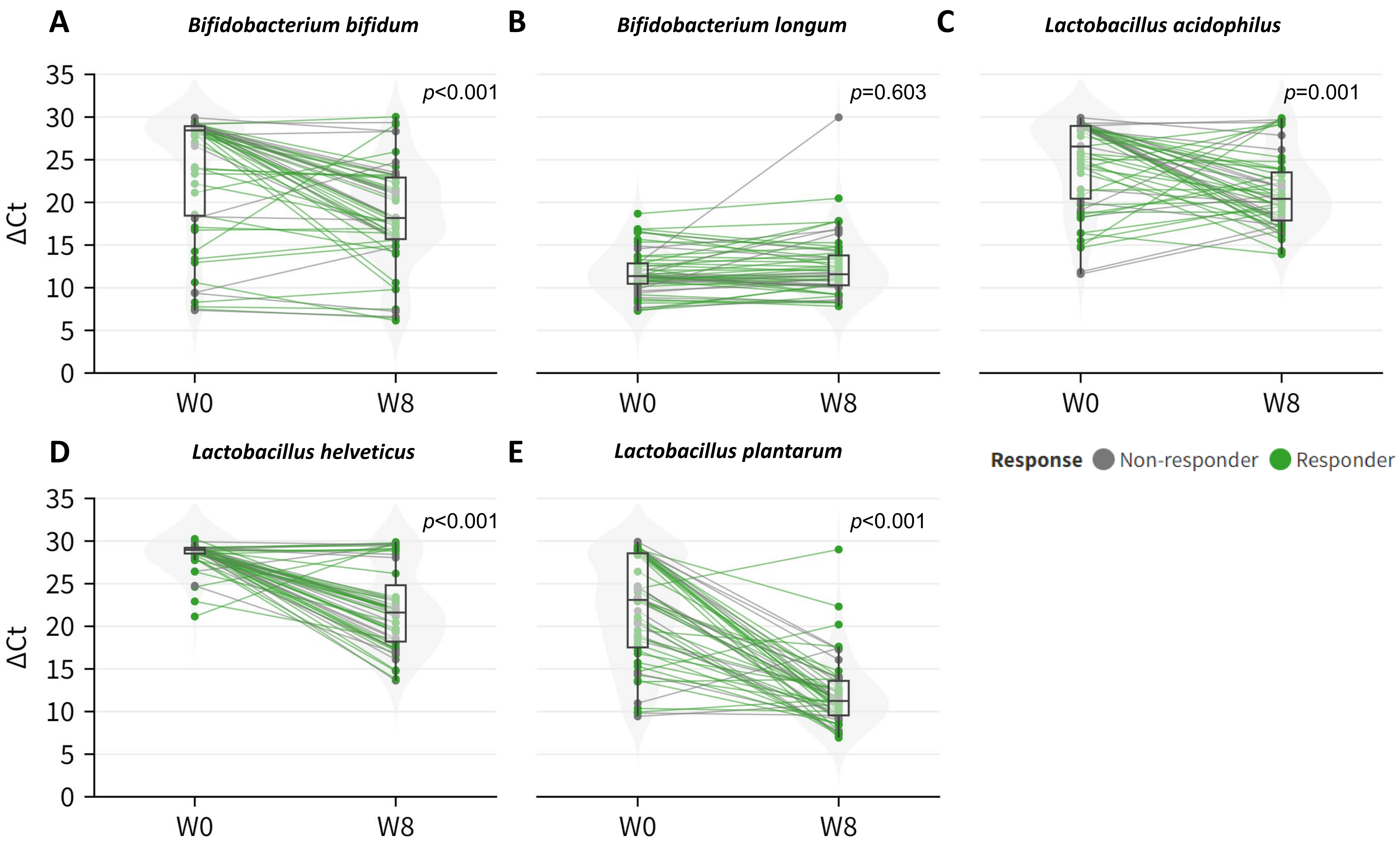

Supplementary Figure S3

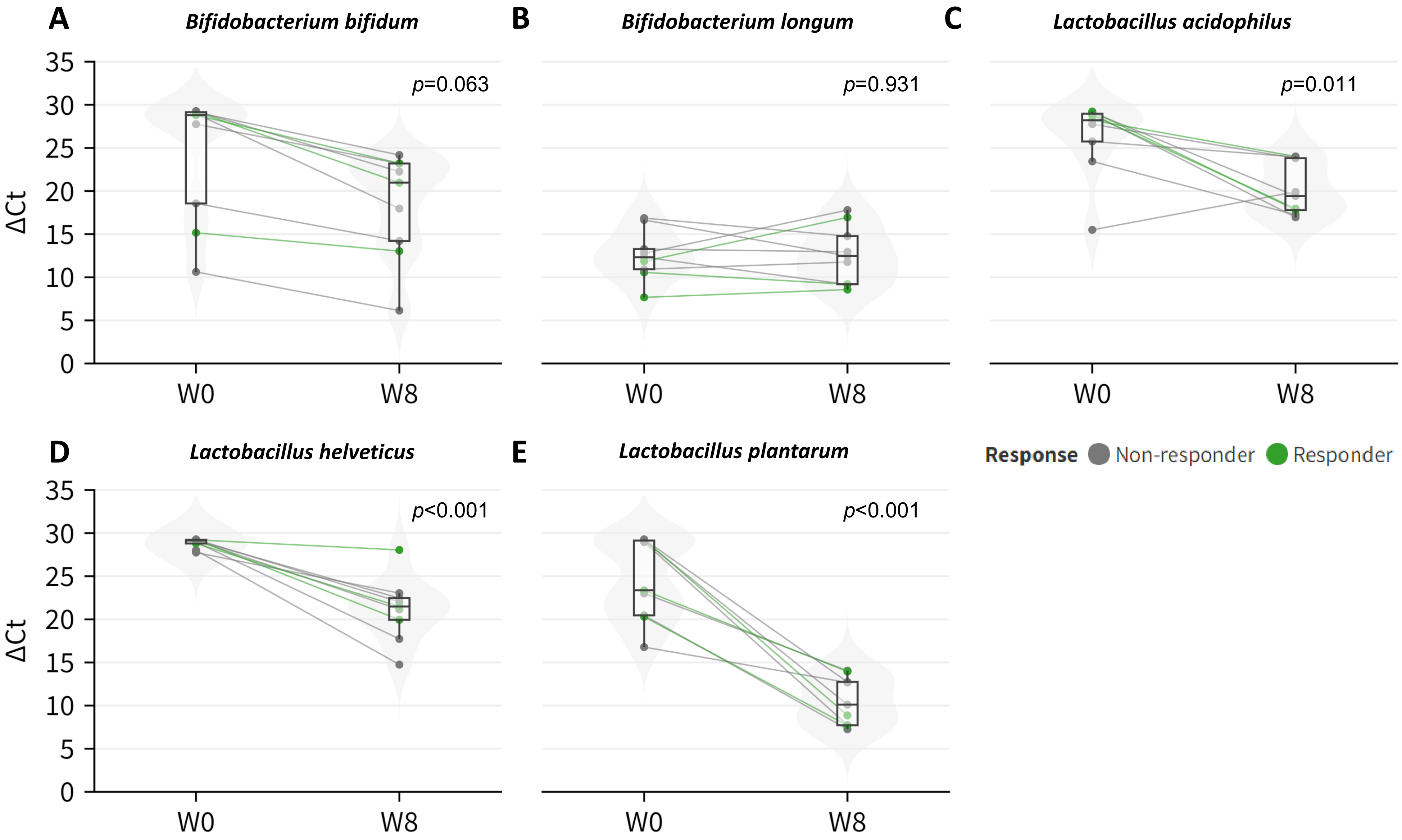

Supplementary Figure S4

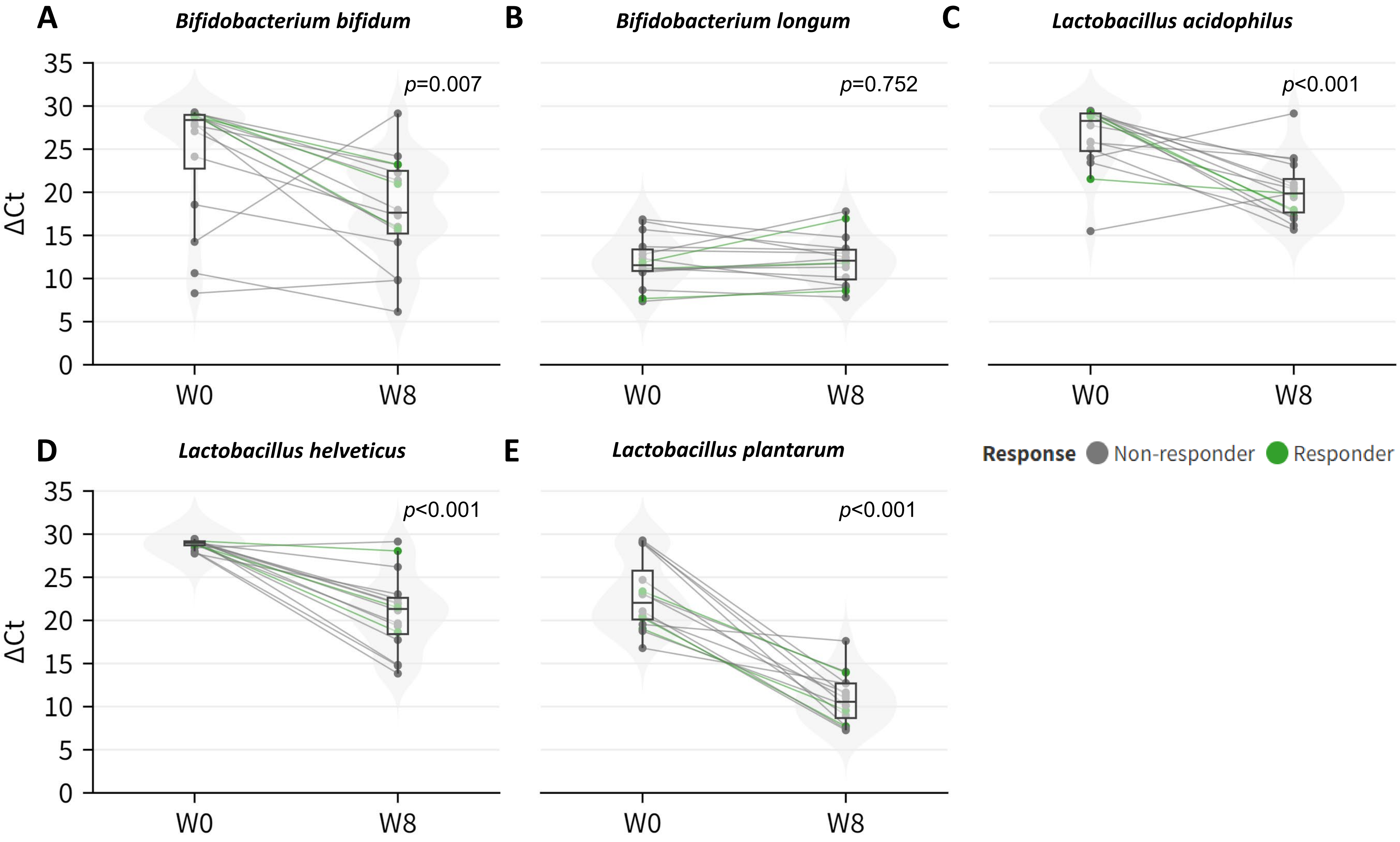

Supplementary Figure S5

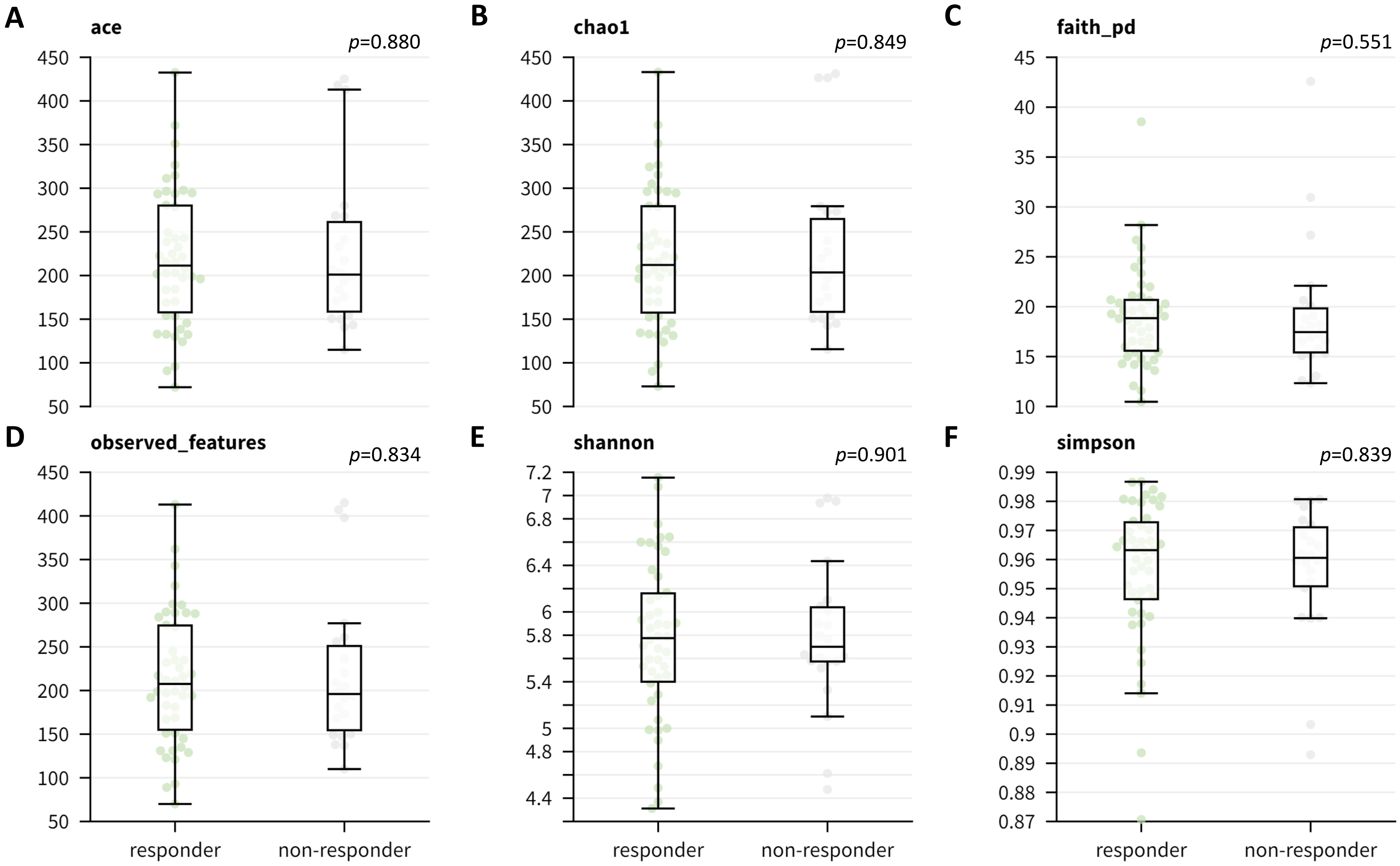

Supplementary Figure S6

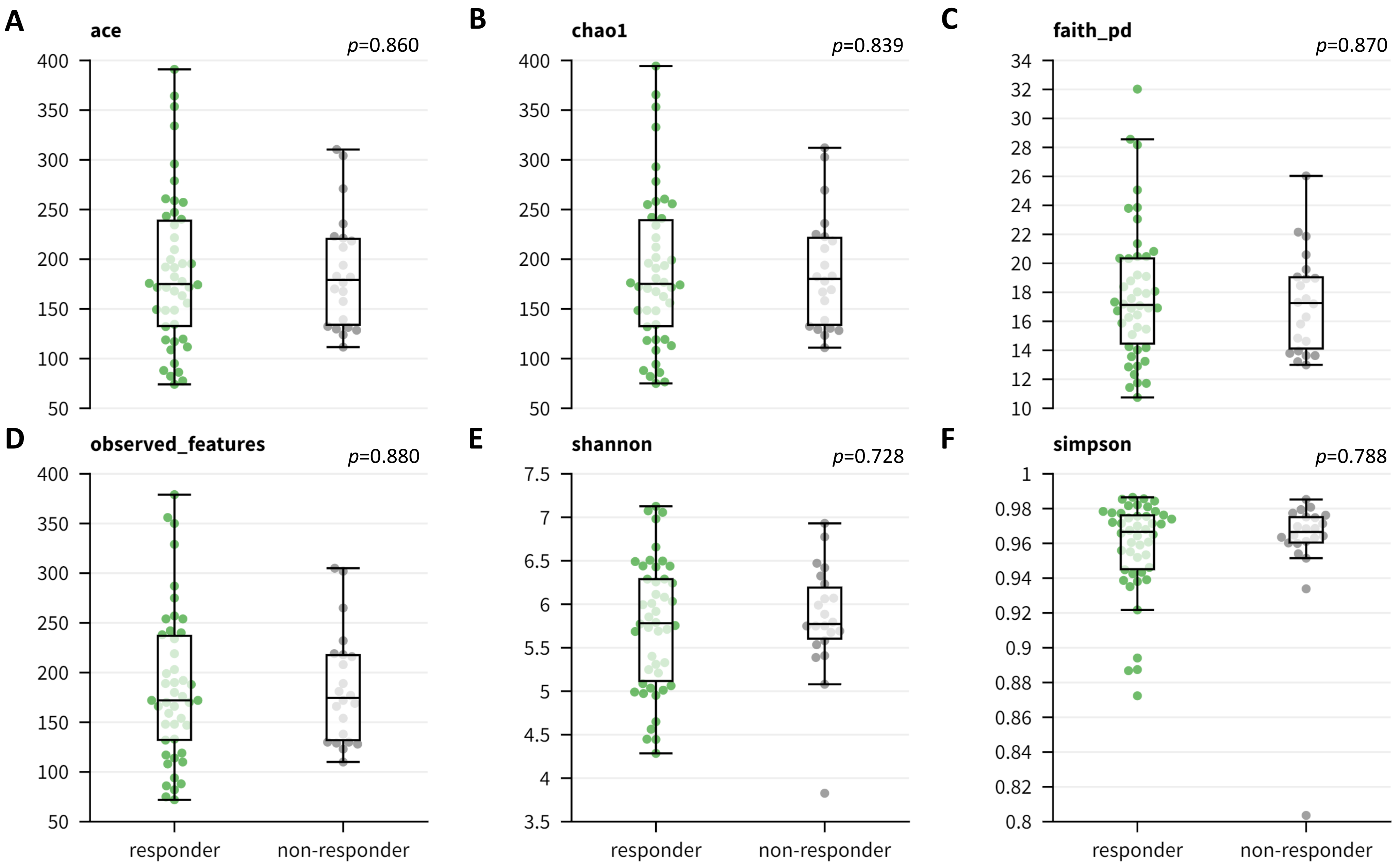

Supplementary Figure S7

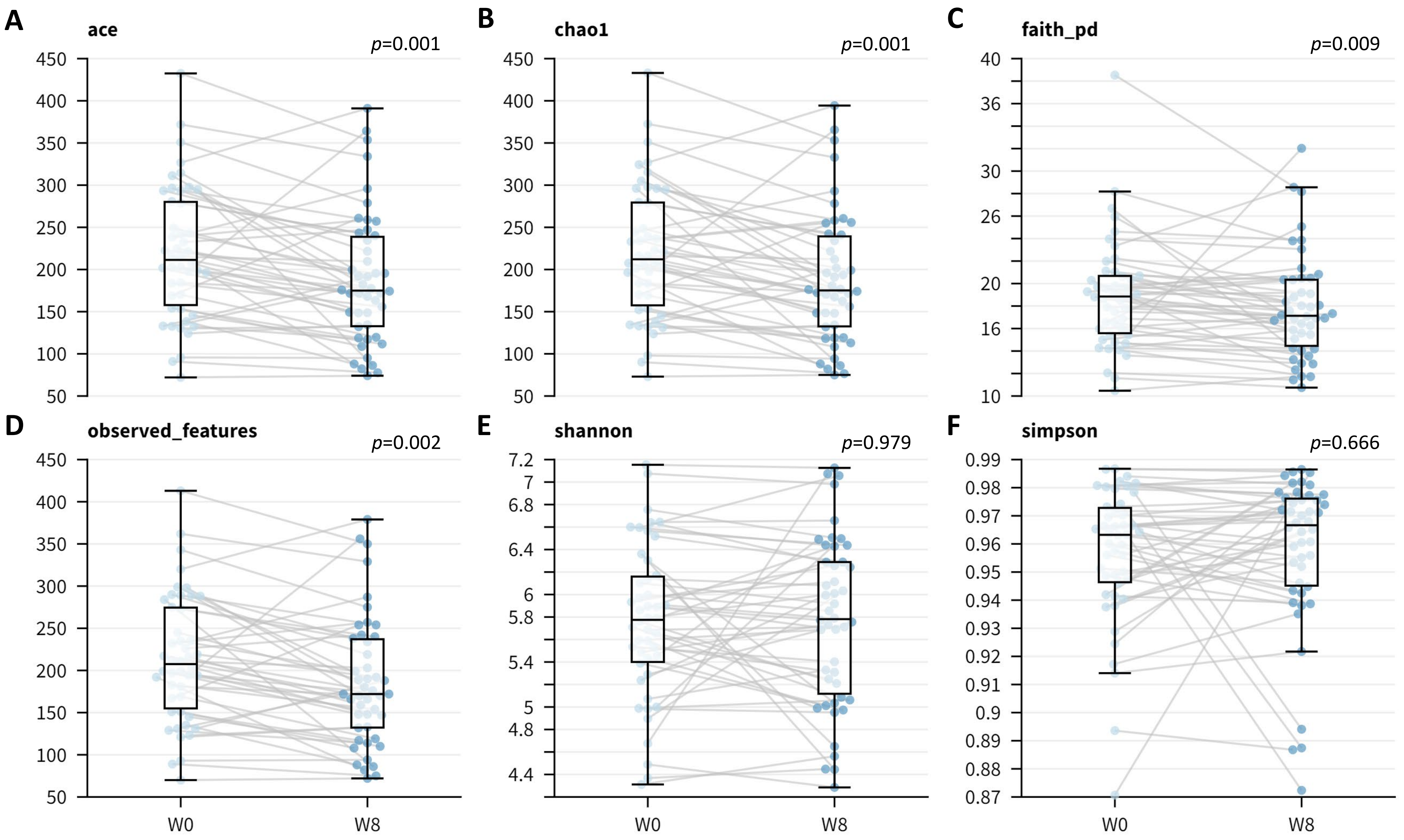

Supplementary Figure S8

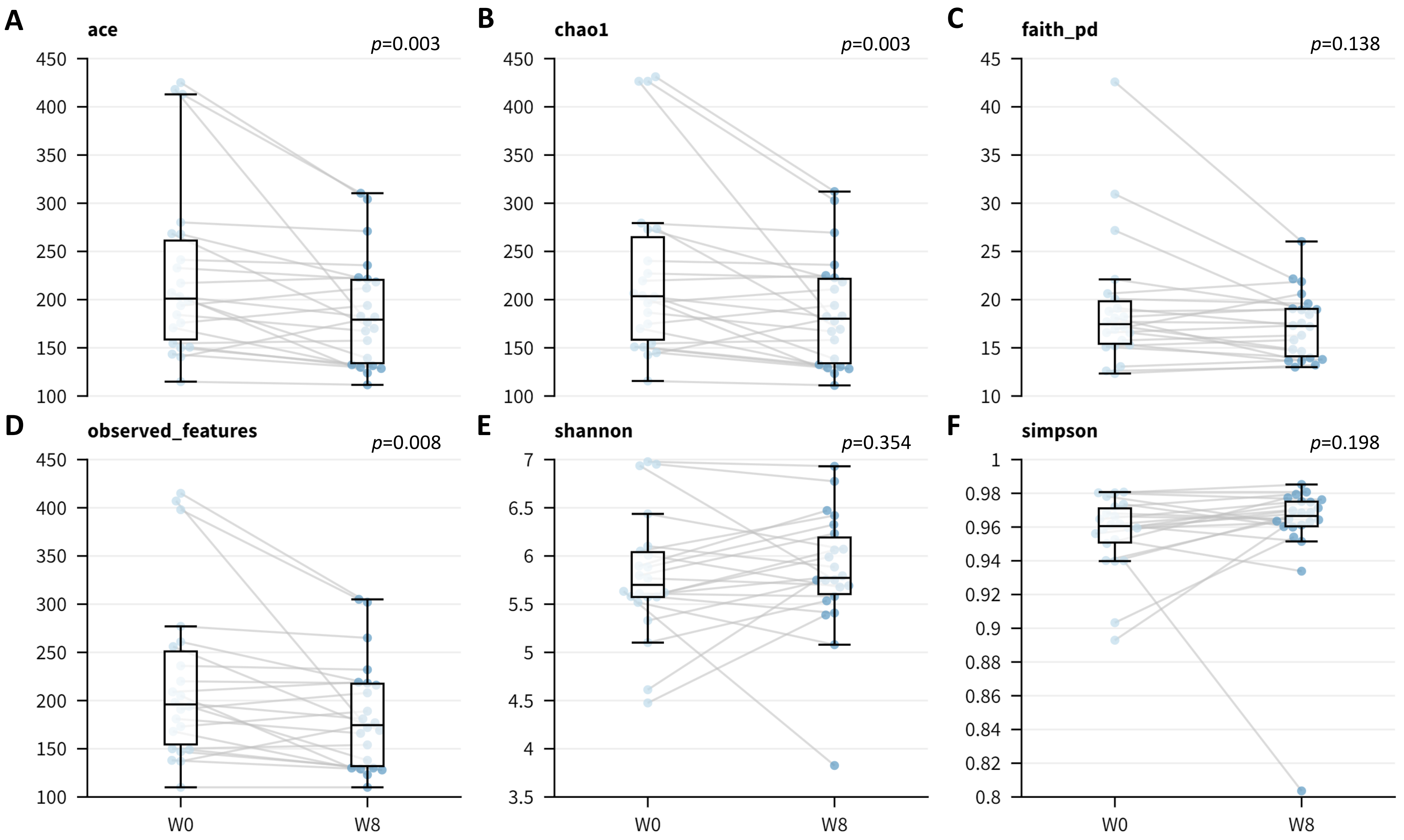

Supplementary Figure S9

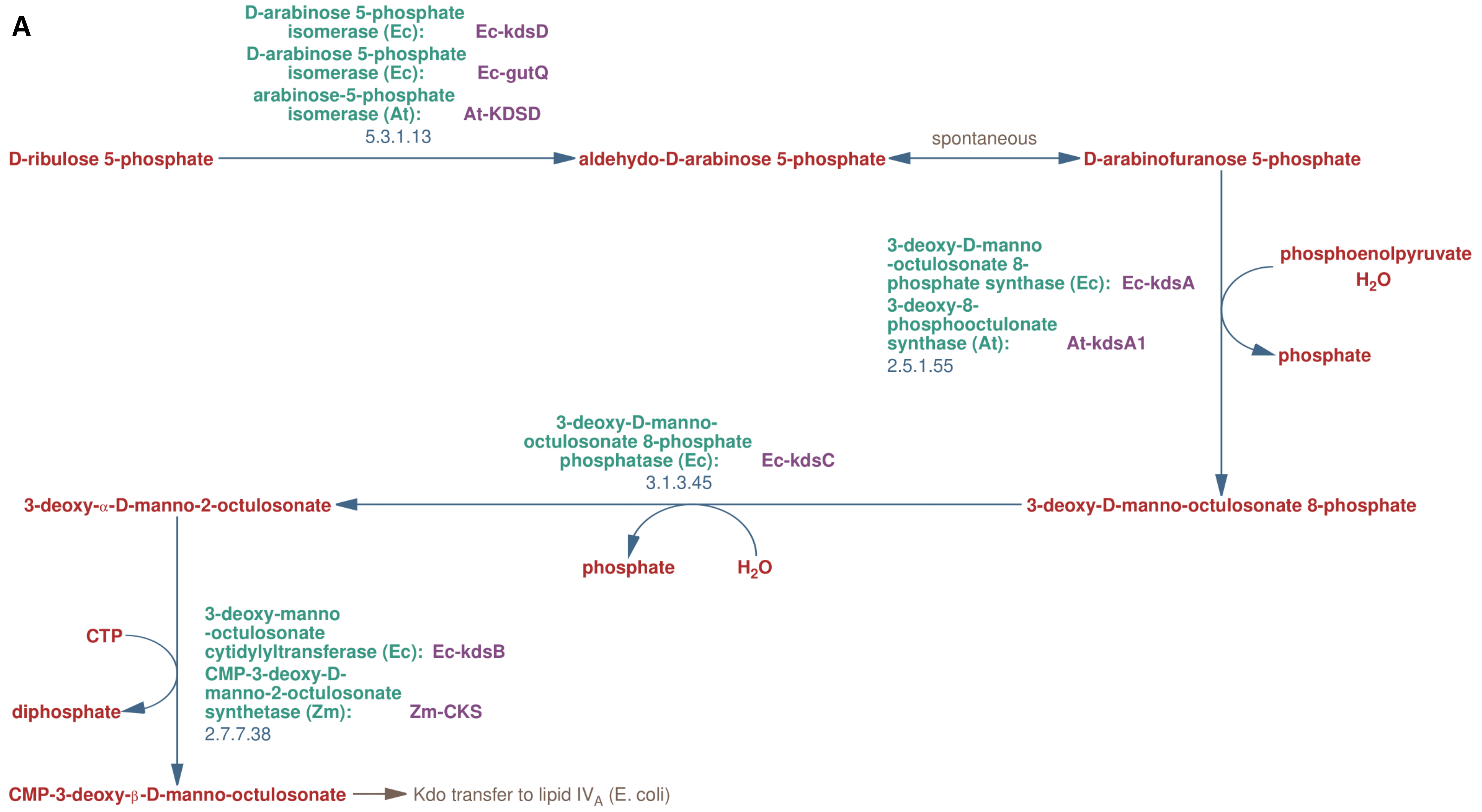

Supplementary Figure S9

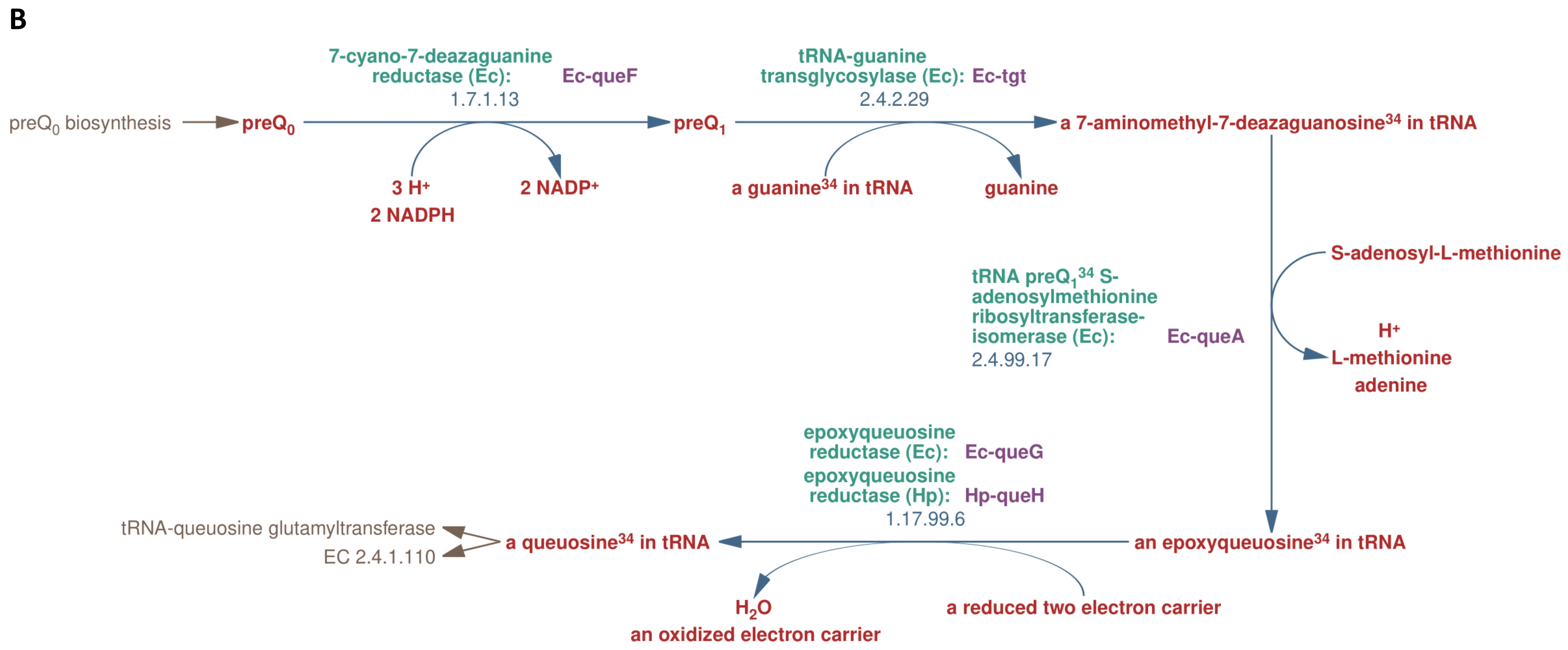

Supplementary Figure S9

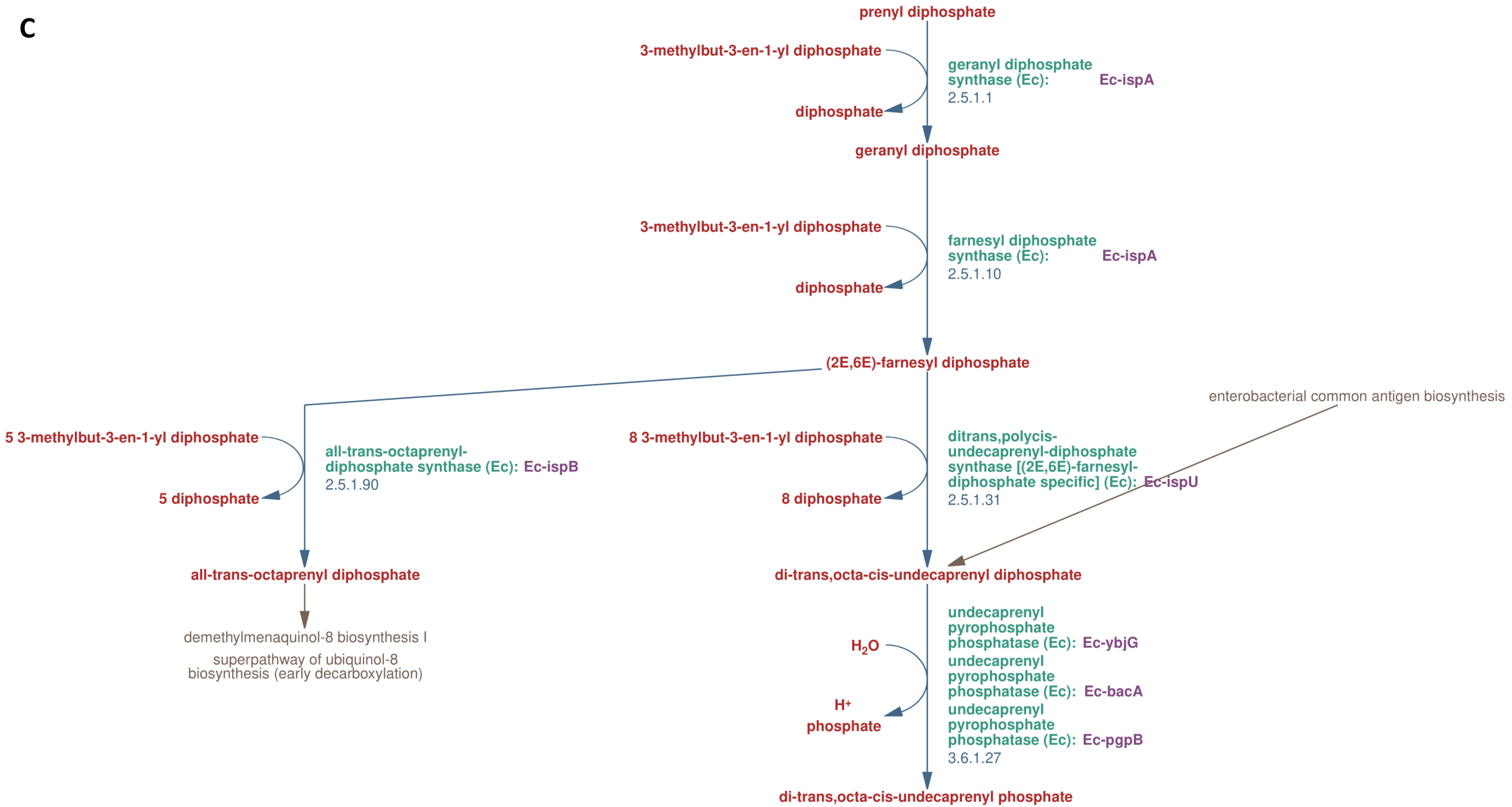

Supplementary Figure S9

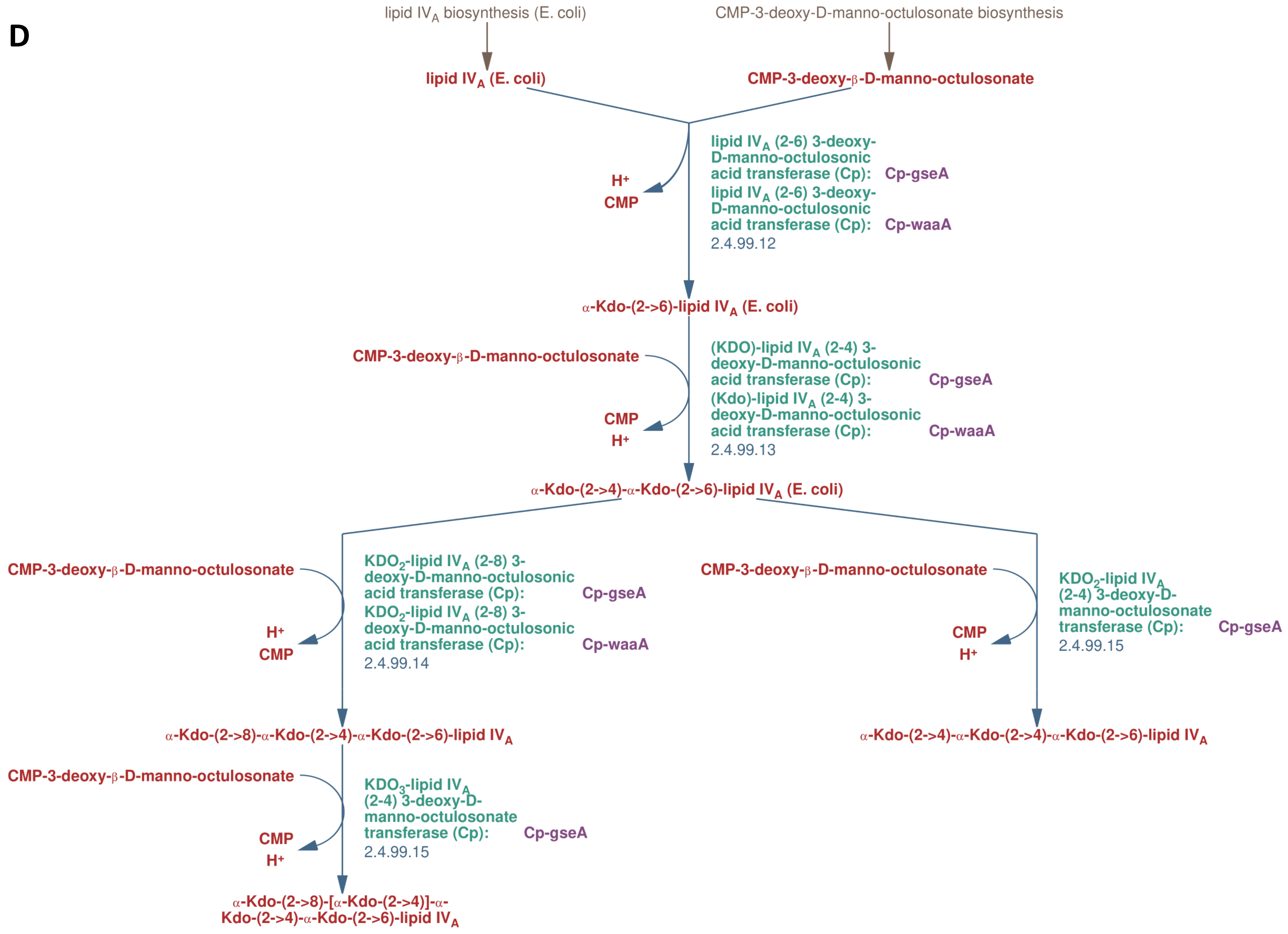

Supplementary Figure S9

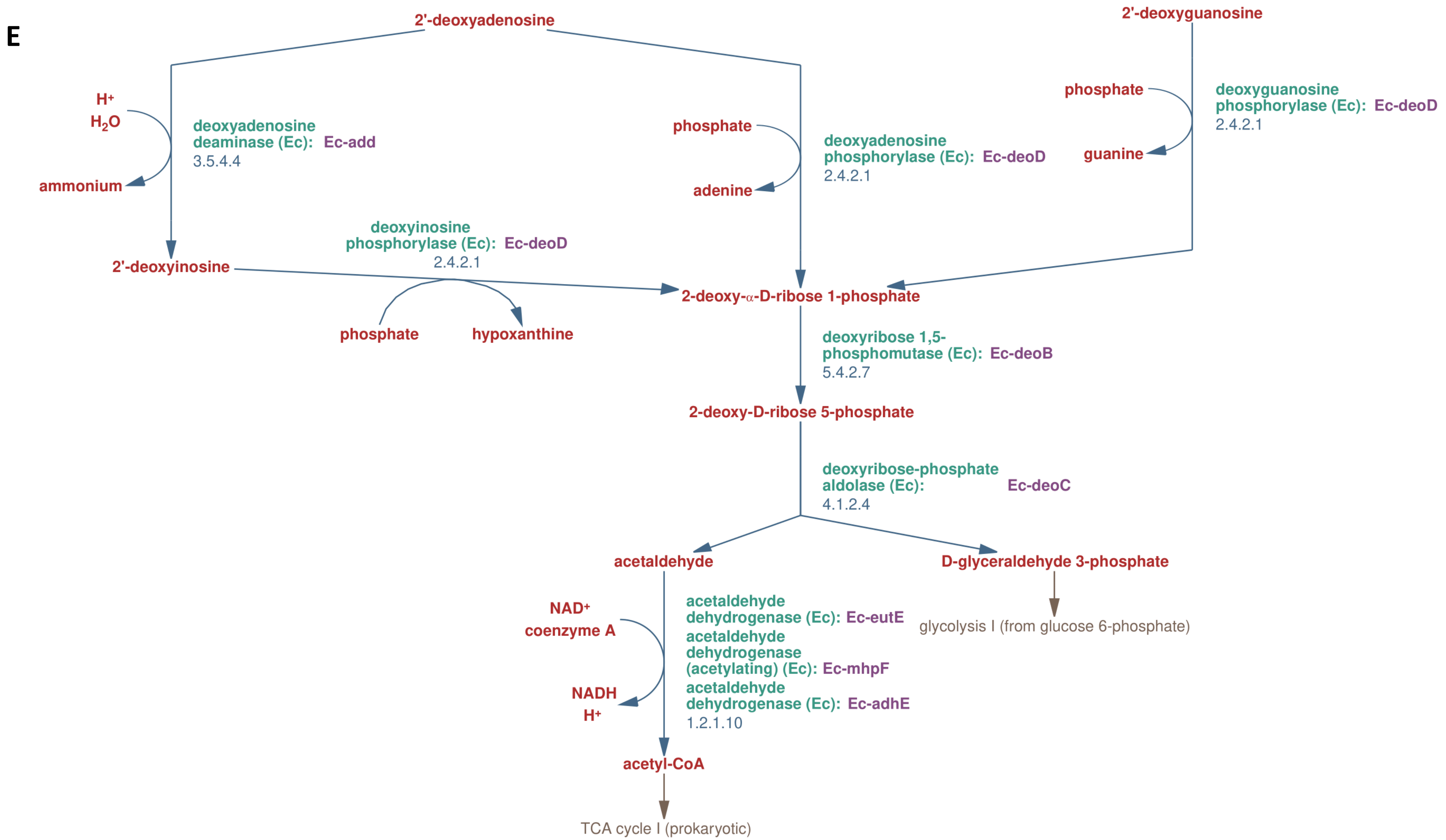

Supplementary Figure S9

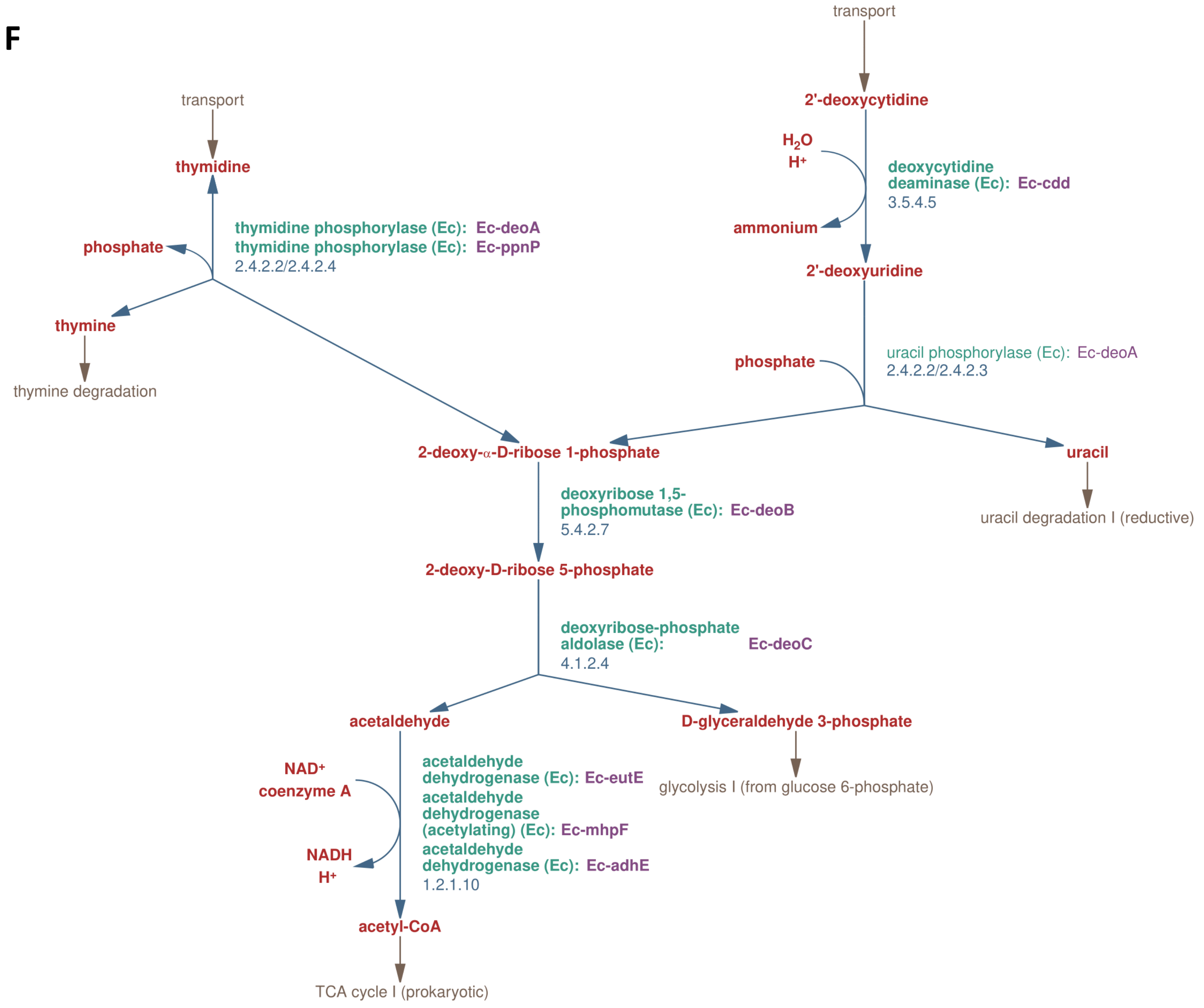

Supplement: Supplementary file 1 [file nutrients-15-05037-s001.zip › mood_suppl-figures_20231205.pdf]
